# Molecular subtypes based on ferroptosis-related genes and tumor microenvironment infiltration characterization in small cell lung cancer

**DOI:** 10.3389/fimmu.2025.1574434

**Published:** 2025-05-13

**Authors:** Xin Wang, Zhenyi Xu, Zhen Lin, Dawei Wu, Yu Tang, Zhihua Pei, Yibo Gao, Jie He

**Affiliations:** ^1^ Department of Clinical Trials Center, National Cancer Center/National Clinical Research Center for Cancer/Cancer Hospital, Chinese Academy of Medical Sciences and Peking Union Medical College, Beijing, China; ^2^ Department of Clinical Trials Center, Shanxi Province Cancer Hospital/Shanxi Hospital Affiliated to Cancer Hospital, Chinese Academy of Medical Sciences/Cancer Hospital Affiliated to Shanxi Medical University, Shanxi, Taiyuan, China; ^3^ Shanghai Key Laboratory of Clinical Geriatric Medicine, Huadong Hospital Affiliated to Fudan University, Shanghai, China; ^4^ Department of Oncology, Zhejiang Provincial People's Hospital, Hangzhou, China; ^5^ Hubei Key Laboratory of Agricultural Bioinformatics, College of Informatics, Huazhong Agricultural University, Wuhan, China; ^6^ Department of Thoracic Surgery, National Cancer Center/National Clinical Research Center for Cancer/Cancer Hospital, Chinese Academy of Medical Sciences and Peking Union Medical College, Beijing, China

**Keywords:** SCLC, molecular subtypes, ferroptosis, TME, immunotherapy

## Abstract

**Background:**

Ferroptosis is an iron-dependent form of regulated cell death associated with cancer. However, the characteristics of ferroptosis in small cell lung cancer (SCLC) are still uncertain. This study aimed to explore the application value of ferroptosis-related genes (FRGs) classification in prognosis and characteristics prediction to provide clues for targeted SCLC therapy.

**Method:**

We systematically characterized mRNA expression and genetic alterations of FRGs in SCLC, evaluating their expression pattern in 181 samples from 3 datasets. Unsupervised clustering analysis was performed to identify the molecular subtypes based on FRGs. We then conducted association analyses between FRG subtypes and various tumor microenvironment (TME) characteristics, traditional key transcript factor subtypes, clinical features, transcriptional and post-transcriptional regulation, drug response, and the efficacy of immunotherapy. Furthermore, the novel classification was validated in an independent cohort of 34 samples from Beijing.

**Result:**

In this study, we identified three distinct ferroptosis subtypes in SCLC: S1, S2, and S3. We found that patients in S2 had the poorest prognosis. The FRG classification was correlated with the NOTCH pathway, MYC pathway, Neuroendocrine (NE), and epithelial-to-mesenchymal transition (EMT) process. Additionally, the FRG classification was strongly associated with TME 4 subtypes. To validate the classification, we employed an independent cohort. The FRG classification could also help to guide the prediction of chemical drugs. Finally, the heatmap showed the landscape of FRG subtypes, TME subtypes, NE subtypes, key transcription subtypes, age, gender, and stage.

**Conclusion:**

Our identification of new SCLC subtypes provides novel insights into tumor biology and has potential clinical implications for the management of SCLC.

## 1 Introduction

Small cell lung cancer (SCLC) is an aggressive and highly lethal type of lung cancer characterized by rapid growth, early metastasis, and resistance to therapy (1). Converging evidence from primary tumors, patient-derived xenografts, cell lines, and genetically engineered mouse models has led to the identification of a novel SCLC subtype classification based on the differential expression of four key transcription factors: ASCL1, NeuroD1, Yap1, and POU2F3 (2). Gay et al. (3) redefined SCLC into four subgroups according to the ASCL1, NEUROD1, POU2F3, and immune-related genes, naming these four subgroups. The SCLC-A and SCLC-N were classified as neuroendocrine subtypes. Conversely, the non-neuroendocrine subtypes encompassed the SCLC-P, SCLC-Y, and SCLC-I. Although the newly characterized SCLC-L subtype has shown potential therapeutic benefit when treated with atezolizumab in combination with chemotherapy, it represents a small fraction of SCLC cases. Therefore, the development of novel and more effective combinatorial therapeutic strategies remains critical to improving the prognostic outcomes of SCLC patients (4, 5).

In recent years, ferroptosis induction has become a promising treatment alternative to trigger cancer cell death, especially for those aggressive malignant tumors resistant to traditional therapies (6, 7). Ferroptosis can be induced through extrinsic or intrinsic pathways (8). The extrinsic pathway is initiated through the regulation of transporters or circular RNA (9). In contrast, the intrinsic pathway is mainly caused by blocking the expression or activity of intracellular antioxidant enzymes, such as glutathione peroxidase 4 (GPX4). The antioxidant enzyme GPX4 can directly reduce phospholipid hydroperoxide to a hydroxy phospholipid, thus acting as a central repressor of ferroptosis in cancer cells (10). Recent studies have reported that shikonin could induce ferroptosis in SCLC and effectively trigger suppressed cell proliferation, apoptosis, migration, invasion, and colony formation (11). Sulforaphane also induced SCLC cell death mediated by ferroptosis (12). Previous studies demonstrated that models based on ferroptosis genes and associated regulatory networks can predict the prognosis and clinical benefit of different treatments in various cancers (13–17). Furthermore, multi-omics analysis facilitates the exploration of the process (18).

Nevertheless, previous research has mainly used cellular and animal experiments to investigate the role of ferroptosis in SCLC, with minimal exploration in human tumor samples. In this study, 221 samples were divided into three subtypes based on 14 FRGs integrating transcriptome and genome. The survival outcomes and various characteristics, such as immune infiltration differences, genomic hallmarks, and drug response across these subtypes, were then explored. Additionally, we employed an independent Beijing cohort to validate the efficacy of FRG-based classification. The findings indicate that FRG-based classification could be a valuable tool for supporting clinical decision-making in oncology.

## 2 Materials and methods

### 2.1 Public cohorts and data process

We collected several publicly available information on transcriptomics, genomics, and clinical data in small cell lung cancer (SCLC). First, we downloaded transcriptomic expression, somatic mutation, and clinical data of SCLC from George et al. (18) (n=81, RNA-seq). The RNA-seq data (Illumina TruSeq) were transformed by log2(FPKM+1). Second, we extracted the expression profile and clinical data of SCLC in GSE60052 (19) (n=79, RNA-seq) and GSE30219 (20) (n=21, Affymetrix) from Gene Expression Omnibus (GEO, https://www.ncbi.nlm.nih.gov/geo/) and Supplementary Materials of papers. Meanwhile, the normal samples were gathered from GSE30219 (n=14). Furthermore, we obtained the expression of genes in SCLC lines from Genomics of Drug Sensitivity in Cancer (GDSC, https://www.cancerrxgene.org) (n=61). Gene expression was illustrated by plotting the median expression value when the genes were with one more probe. The intersect genes present in George’s Cohort, GSE60052, GSE30219, and SCLC lines in GDSC were combined into an overall dataset. Batch effects from non-biological technical biases were corrected using the “ComBat” algorithm of the “sva” package (21). Furthermore, genes with more than 50% equal to 0 value were deleted. The copy number variation (CNV) data of SCLC lines were retrieved from the Broad Institute Cancer Cell Line Encyclopedia (https://depmap.org/portal/download/) (22). Copy number data were log2 transformed with a pseudo-count of 1. A total of 56 SCLC cell lines were overlapped between GDSC and CCLE for further CNV analysis. We used<0.5 as the threshold for copy number loss, and >1.5 as the threshold for copy number gain (23).

### 2.2 Patient selection and recruitment

The study protocol conformed to the ethical guidelines of the Declaration of Helsinki (as revised in 2013). The study protocol and informed consent in this study were reviewed and approved by The Medical Ethics Committee of Cancer Hospital, Chinese Academy of Medical Sciences (approval no. 23/020-3759) and individual consent for this retrospective analysis was waived.

We retrospectively collected surgically resected, formalin-fixed, paraffin-embedded SCLC samples from the biobank of the National Cancer Center/National Clinical Research Center for Cancer/Cancer Hospital in Chinese Academy of Medical Sciences and Peking Union Medical College (Beijing, China). Thirty-four pathologically and clinically diagnosed SCLC patients were recruited from 2010 to 2013 as an independent cohort and the clinical data were obtained by reviewing the patients' medical histories, which are summarized in **Supplementary Table S1**.

### 2.3 RNA extraction and sequencing

Sequencing was performed using a NovaSeq 6000 S4 following Illumina-provided protocols for 2x150 paired-end sequencing in Mingma Technologies (Shanghai, China). After FFPE sample sections were scalpeled into 1.5 mL micro centrifuge tube, deparaffinization solution was used to remove paraffin. Then Maxwell 16 LEV RNA FFPE kit (Promega) was used to extract FFPE RNA according the protocol’s instructions. The captured coding regions of the transcriptome from total RNA were prepared using TruSeq® RNA Exome Library preparation Kit. The cDNA was generated from the input RNA fragments using random priming during first and second strand synthesis and sequencing adapters were ligated to the resulting double-stranded cDNA fragments. The coding regions of the transcriptome were then captured from this library using sequence-specific probes to create the final library. After the library was constructed, Qubit 2.0 fluorometer dsDNA HS Assay (Thermo Fisher Scientific) was used to quantify concentration of the resulting sequencing libraries, while the size distribution was analyzed using Agilent BioAnalyzer 2100 (Agilent). The RNA-seq data (Illumina TruSeq) were transformed log2(FPKM+1) and gene expression was illustrated by plotting the median expression value when the genes were with one more probe.

### 2.4 Ferroptosis-related consensus clustering analysis

We gathered 266 ferroptosis genes from FerrDb (http://www.zhounan.org/ferrdb/) (24) and recently published research (25, 26) (**Supplementary Table S2**), and a total of 221 overlapped genes from different detection platforms were extracted by combined with public cohorts for further analysis. Unsupervised clustering analysis was conducted to identify distinct ferroptosis-subtypes based on the expression profiles of 221 ferroptosis-based genes in batch correction dataset. Before unsupervised clustering, the correction dataset was preprocessed by median centering. The “ConsensuClusterPlus R package (27) was applied to execute the consensus clustering via hierarchical cluster, 1,000 times repetitions and resample rate of 80% were performed for stable clustering. Furthermore, we compared the SCLC subtypes defined by relative expression of four transcription regulators proposed by Rudin et al. (2) and ferroptosis subtypes to profile and supplement SCLC characteristics. NCC Cohort was used to validate the characteristics of ferroptosis subtypes.

### 2.5 Identification of differentially expressed genes associated with ferroptosis subtypes and enrichment analysis

The differentially expressed genes (DEGs) and differentially expressed ferroptosis-related genes (DEFGs) among ferroptosis subtypes were screened out using the R package “limma” (28). The absolute value of log2 fold change (FC) larger than 1 and Benjamini–Hochberg (BH) adj P-value< 0.05 were taken as significance criteria. To further infer the biological functions and signals involving the DEGs, the enrichment analysis of the Kyoto Encyclopedia of Genes and Genomes (KEGG) pathway and Gene Ontology (GO) function were performed by “clusterProfiler” (29) R package, with the cutoff value of BH adj P< 0.05. To investigate the important functional phenotypes among different ferroptosis subtypes, we performed gene set variation analysis (GSVA) (30) and gene set enrichment analysis (GSEA) (31). The “h.all.v7.5.symbols.gmt” gene set was used as the reference gene sets and obtained from the MSigDB database (https://www.gsea-msigdb.org/gsea/msigdb/). In GSEA, normalized enrichment score (NES) > 1.5 and false discovery rate (FDR) P< 0.05 were regarded as significant enrichment.

### 2.6 Estimation of the tumor microenvironment in SCLC

TME plays as a significant role in clinical outcomes and response to therapy. We described the relative abundance of 29 functional gene expression signatures in SCLC by introducing the single sample gene set enrichment analysis (ssGSEA) algorithm. The signatures were derived from the study of Bagaev.A et al. (32). The enrichment score derived from ssGSEA reflects the relative degree of TME in each patient.

### 2.7 The quantification of EMT score

The epithelial-to-mesenchymal transition (EMT) is a critical cell biological process that occurs during cancer development. In our study, we assessed EMT gene signature, including 25 epithelial and 52 mesenchymal marker genes (33, 34). The EMT score of each sample was calculated with formula $\sum_{i}^{N}\frac{M^{i}}{N}-\sum_{j}^{n}\frac{E^{j}}{n}$ . Here, M and E represent the expression of mesenchymal and epithelial marker genes, N and .. represent the gene number of mesenchymal marker and epithelial marker, respectively.

### 2.8 The quantification of NE score

SCLC is a high grade neuroendocrine (NE) tumor, while a subset of SCLC has been described “variant” due to the loss of NE characteristics and exhibited more aggressive growth. Using the 50 gene NE signature to quantify the NE score, ranging from -1 to 1, with a more positive score indicating higher NE properties (35). NE score was generated by the formula: NEscore = (correl NE-correl >non-NE)/2 , where correl NE (or non-NE) is the Pearson correlation coefficient between expression of the 50 genes in the test sample and expression of these genes in the NE (or non-NE) cell line group.

### 2.9 Evaluation of key characteristics and immunecheckpoint

We extracted tumor cell characteristics and subclonal architecture of SCLC in George’s Cohort (12), including purity, ploidy, cancer cell fraction (CCF) per read values, and CCF First Subclone. Intratumor heterogeneity (ITH) was estimated by MATH score (36, 37). In our study, we obtained “Allelic_Fraction_Tumor” and calculated MATH score in George’s Cohort. And tumor mutational burden (TMB) was quantified by mutation count (excluding nonsense mutation) in samples, and transformed by log2. Estimation of Stromal and Immune cells in Malignant Tumors using Expression data (ESTIMATE) (38) was applied to quantify the infiltration of stromal and immune components in tumor, reflecting the tumor microenvironment. Tumor Immune Dysfunction and Exclusion (TIDE, http://tide.dfci.harvard.edu/), a computational method to predict immune checkpoint blockade response, was developed by Jiang et al. (39). TIDE integrates tumor immune evasion mechanisms including T cell dysfunction and T cell exclusion, and has shown superior predicted performance of immunotherapy response. Furthermore, the mRNA expression of immune checkpoints was analyzed in different ferroptosis subtypes.

### 2.10 Drug sensitivity prediction

To discover potentially sensitive drugs, the chemotherapeutic response among ferroptosis subtypes was calculated in GDSC SCLC lines. Comparison among ferroptosis subtypes was analysis by Kruskal-Wallis in half-maximal inhibitory concentration (IC50) to each chemotherapy drug. To predict the response of ferroptosis subtypes to immunotherapy in SCLC, the subclass mapping (SubMap) algorithm was employed for further verification (40). The analysis estimated the similarity of gene expression profiles between SCLC subgroups and a cohort of 47 melanoma patients treated with anti-PD-1 and anti-CTLA-4 immune therapy (41). If the gene expression profiles showed significant similarity, patients in the subtype were inferred to be likely sensitive to immunotherapy.

### 2.11 Statistical analysis

In this study, all data processing was done in the R-3.6.0 software. Continuous variables were summarized as mean ± SD and categorized variables were described by frequency (n) and proportion (%). Comparison between two groups was calculated using Wilcoxon rank-sum test, while three group comparison was estimated by Kruskal-Wallis test. Principal component analysis (PCA) was performed to visualize the difference of ferroptosis-related genes between tumor and normal samples. Kaplan-Meier curve analysis to generate the survival curves and determined the significance of the differences via the log-rank test. Multivariate Cox regression analysis was used to assess the independent prognostic factor. The “GenVisR” (42). R package was used to depict the mutation and CNV landscape. All statistical test was two-side and P< 0.05 was considered statistically significant. NS: not significant; *P< 0.05; **P< 0.01; ***P< 0.001.

## 3 Results

### 3.1 Landscape of genetic variation of FRGs in SCLC

**Figure 1:**
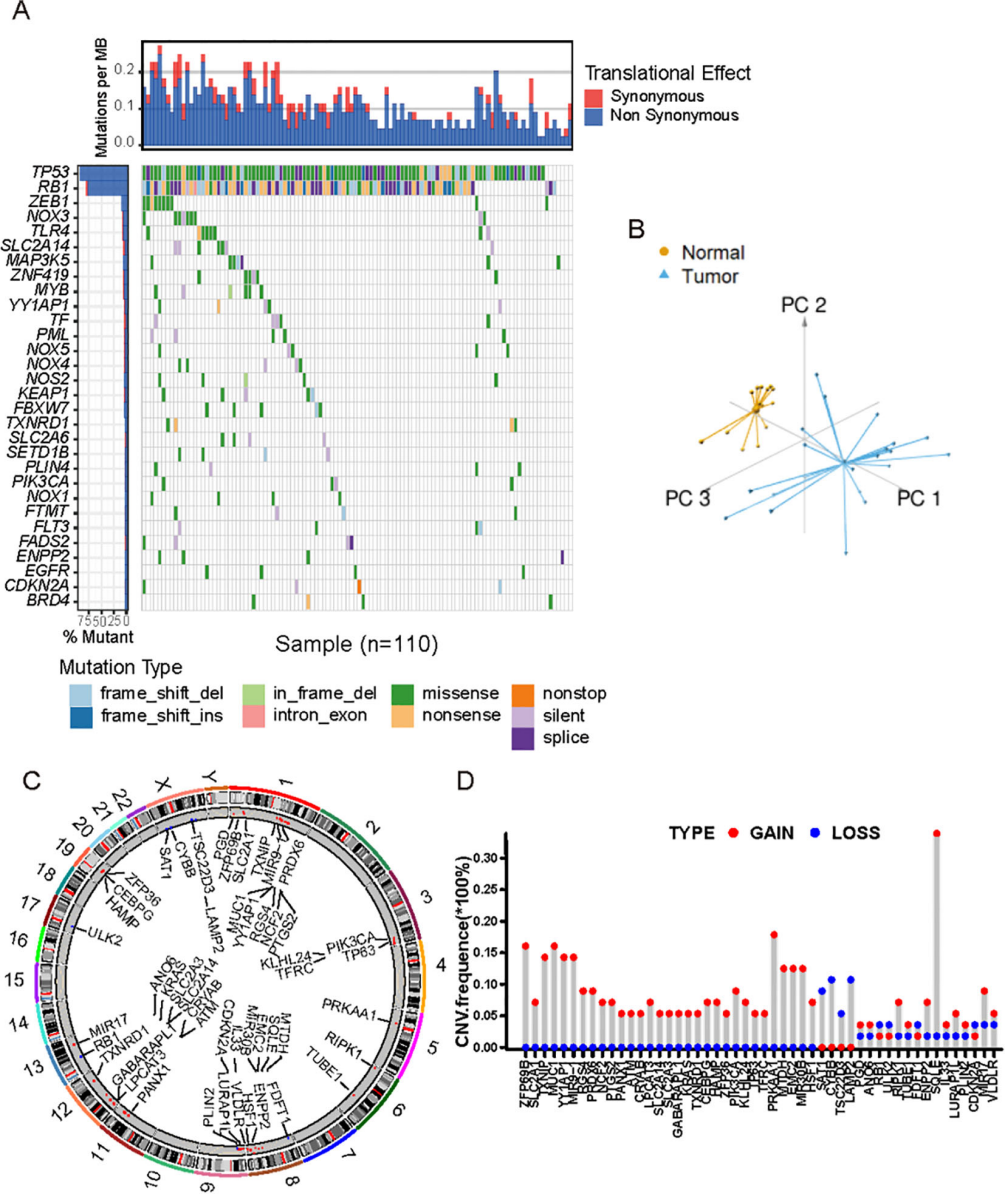
Landscape of genetic and transcriptional characteristics of FRGs in SCLC. **(A)** Waterfall plot shows the mutation distributions of FRGs in SCLC patients from public cohort. Each column represented individual patients. The upper bar plots showed overall number of somatic mutations. The below stacked bar plots displayed the type and fractions of base-pair substitutions of each sample. The numbers on the right indicated the mutation frequency in each gene. The right bar plot showed the proportion of each variant type. **(B)** Principal component analysis showed the tumors were well distinguished from normal samples based on the expression profiles of 211 FRGs. Tumors were marked with blue and normal samples with yellow, respectively. **(C)** The location of CNVs alterations of FRGs on chromosomes. **(D)** The distribution of CNVs of FRGs. The height of the column indicates ed the alteration frequency. Red dot indicates CNV gain, and blue dot CNV loss.

A total of 221 FRGs were identified in this study and the workflow of this research was shown in **Supplementary Figure S1**. First, we summarized the incidence of copy number variations (CNVs) and somatic mutations of 30 FRGs in SCLC. Among 181 samples, 110 mutations of FRGs were revealed with a frequency of 60.8% (**Figure 1A**). Based on the expression of these 221 FRGs, we were able to completely distinguish SCLC samples from normal samples (**Figure 1B**). The CNV locations of FRGs on chromosomes are shown in **Figure 1C**. Analysis of CNV alteration frequencies revealed widespread CNV alterations in 21 regulators, most of which involved amplifications in copy number. However, SAT1, CYBB, TSC22D1, and LAMP2 exhibited frequent CNV deletions (**Figure 1D**). The analyses presented herein demonstrate the heterogeneity of the genetic and expressional alteration landscape in ferroptosis-related genes between normal and SCLC samples, indicating that the expression imbalance of ferroptosis plays a crucial role in the occurrence and progression of SCLC.

### 3.2 Ferroptosis medicated patterns were constructed by FRGs

**Figure 2:**
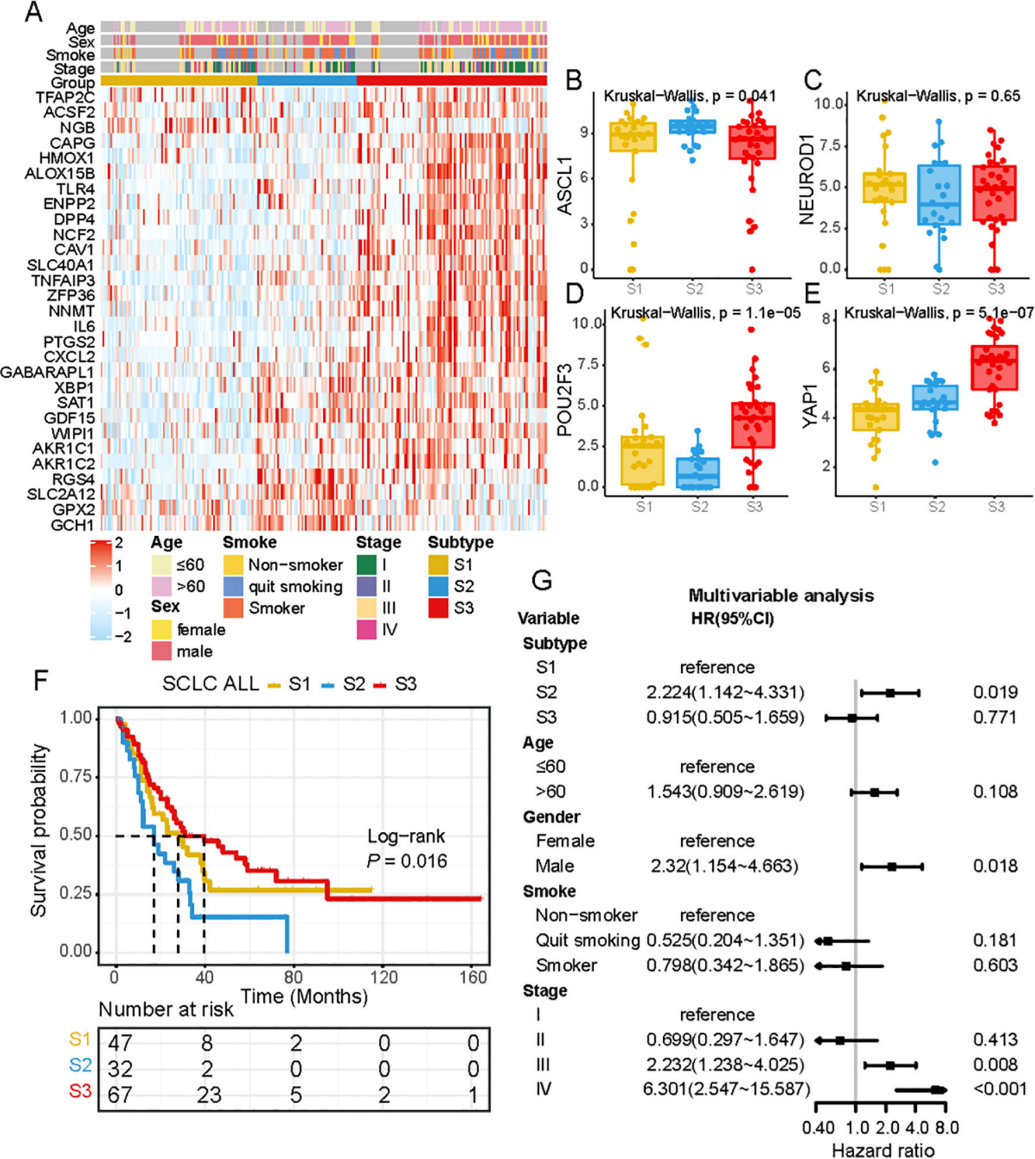
Ferroptosis-related molecular patterns with distinct prognosis, biological characteristics in SCLC. **(A)** Differential expression of NMF-selected genes. **(B-E)** the expression of ASCL1, NEUROD1, POU2F3 and YAP1 across 3 subtypes. **(F)** Survival analysis of three ferroptosis subtypes in all patients, Kaplan-Meier survival curve showed significant differences among the three subtypes (P =0.016, Log-rank test). **(G)** Multivariate Cox regression analysis of clinicopathological factors and ferroptosis subtypes for overall survival in patients across multiple centers.

Unsupervised clustering of 221 ferroptosis-related genes (FRGs) in the SCLC cohort classified patients into three distinct molecular patterns: S1, S2, and S3. The ferroptosis profiles of these subtypes were significantly different (**Figure 2A**). S1 exhibited low expression of FRGs, S2 showed high expression of RGS4, SLC12A2, GPX2, and GCH1, while S3 was characterized by increased expression of most FRGs (**Figure 2A**). The ferroptosis subtypes were classified according to the traditional subtypes defined by four key transcription regulators, including ASCL1, NEUROD1, POU2F3, and Yap1. To further investigate the expression patterns of these regulators within the distinct subtypes, we conducted an independent evaluation (**Figure 2B-E**). Our findings revealed that ASCL1 exhibited high expression in S2, while POU2F3 and Yap1 demonstrated high expression in S3. Furthermore, survival analysis revealed significant differences in prognosis among the three ferroptosis subtypes, with S3 showing significant survival advantages (**Figure 2F**). To evaluate whether ferroptosis subtypes could serve as independent prognostic factors, we performed multivariate Cox regression analysis, adjusting for clinical characteristics such as age, gender, and TNM status. The results revealed that ferroptosis subtypes were robust, independent prognostic biomarkers for assessing patient outcomes (**Figure 2G**). The FRG-based molecular subtyping effectively predicted patient outcomes in the SCLC cohort.

### 3.3 Diverse characteristics of FRGs subtypes

**Figure 3:**
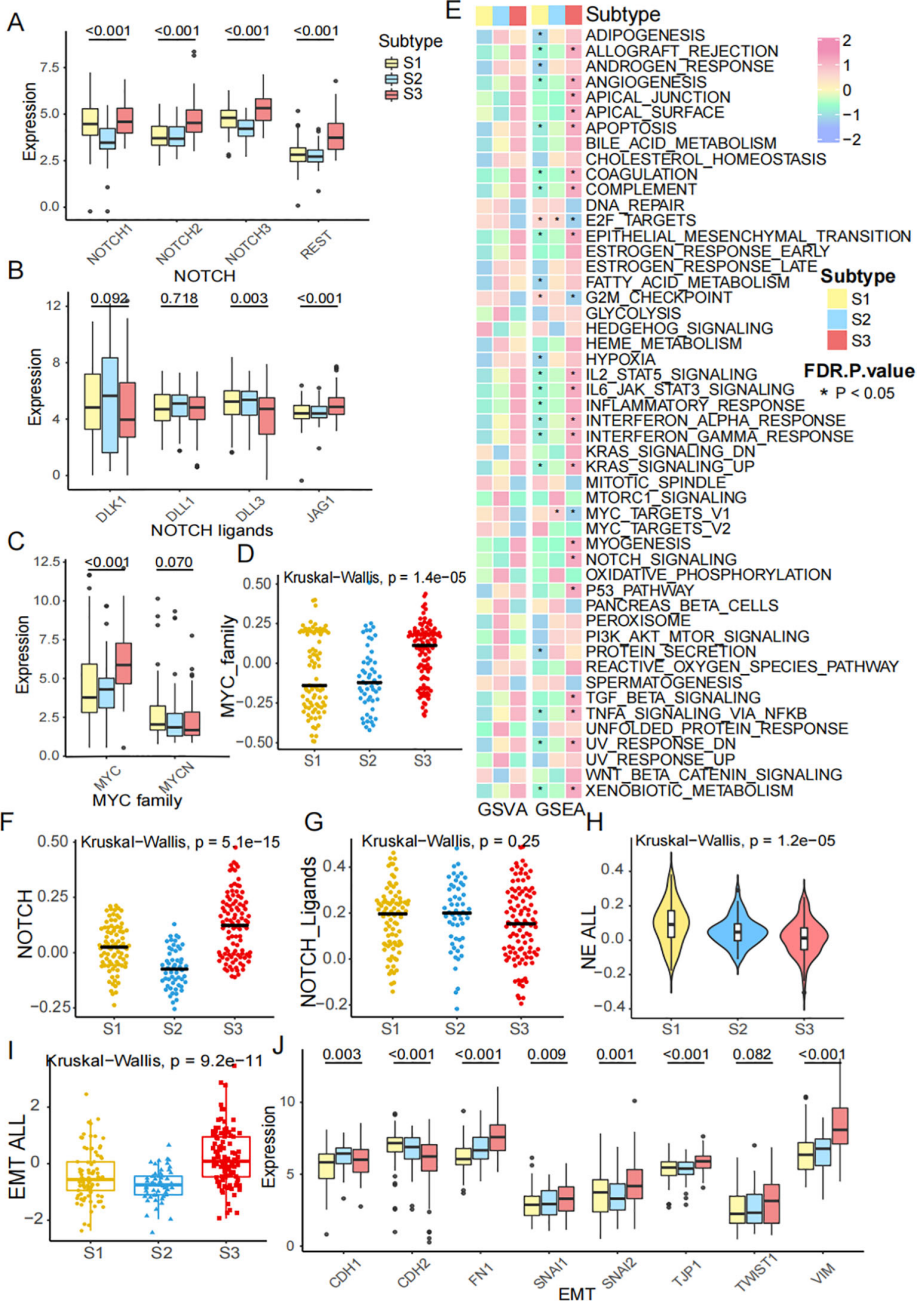
Diverse characteristics of FRGs subtypes. **(A)** The expression of NOTCH1, NOTCH2, NOTCH3 and REST across subtypes. **(B)** The expression of NOTCH ligands, including DLK1, DLL1, DLL3 and JAG1 across subtypes. **(C)** The expression of MYC family, including MYC, MYCN across subtypes. **(D)** MYC pathway score was calculated by ssGSEA across subtypes. **(E)** A heatmap visualizing the GSVA and GSEA enrichment analysis of representative Hallmark pathways shows the activation states of biological pathways in distinct ferroptosis clusters. Red represented activated pathways, and blue represented inhibited pathways. **(F, G)** NOTCH score and NOTCH ligands score across subtypes. **(H)** NE score across subtypes. **(I)** EMT score across subtypes. **(J)** The mRNA expression of EMT genes, including CDH1, CDH2, FN1, SNAI1, SNAI2, TJP1, TWIST1 and VIM.

Our study showed that the characteristic genes in the NOTCH pathway (including NOTCH1, NOTCH2, NOTCH3, and REST) and NOTCH ligands (including DLK1, DLL1, DLL3, and JAG1) were associated with the FRG subtypes (**Figures 3A, B**). Additionally, the MYC and MYCN genes, which are in the MYC pathway, were identified as being correlated with FRG subtypes (**Figure 3C**). To further explore the expression of these genes, we calculated the activity of the MYC pathway using ssGSEA, which suggested that the MYC pathway could effectively distinguish the subtypes (**Figure 3D**). A heatmap visualizing the GSVA and GSEA enrichment analysis of representative Hallmark pathways shows the activation states of biological pathways in distinct FRG subtypes (**Figure 3E**). Both GSEA and GSVA revealed similar activated pathways in S3. Meanwhile, the NOTCH pathway was significantly upregulated in S3, while the NOTCH ligands pathway was not (**Figures 3F, G**). Neuroendocrine (NE) differentiation has been recognized as a specific characteristic of SCLC. We calculated the NE score and found it to be lower in S3 (**Figure 3H**) and the activity of the epithelial-mesenchymal transition (EMT) process was the highest (**Figure 3I**). The genes involved in the EMT process are shown in the boxplot, which is consistent with the previous results (**Figure 3J**). These findings indicated that the FRG classification was correlated with the NOTCH pathway, MYC pathway, NE differentiation, and the EMT process.

### 3.4 The FRGs classification is tightly correlated with TME 4 subtypes

**Figure 4:**
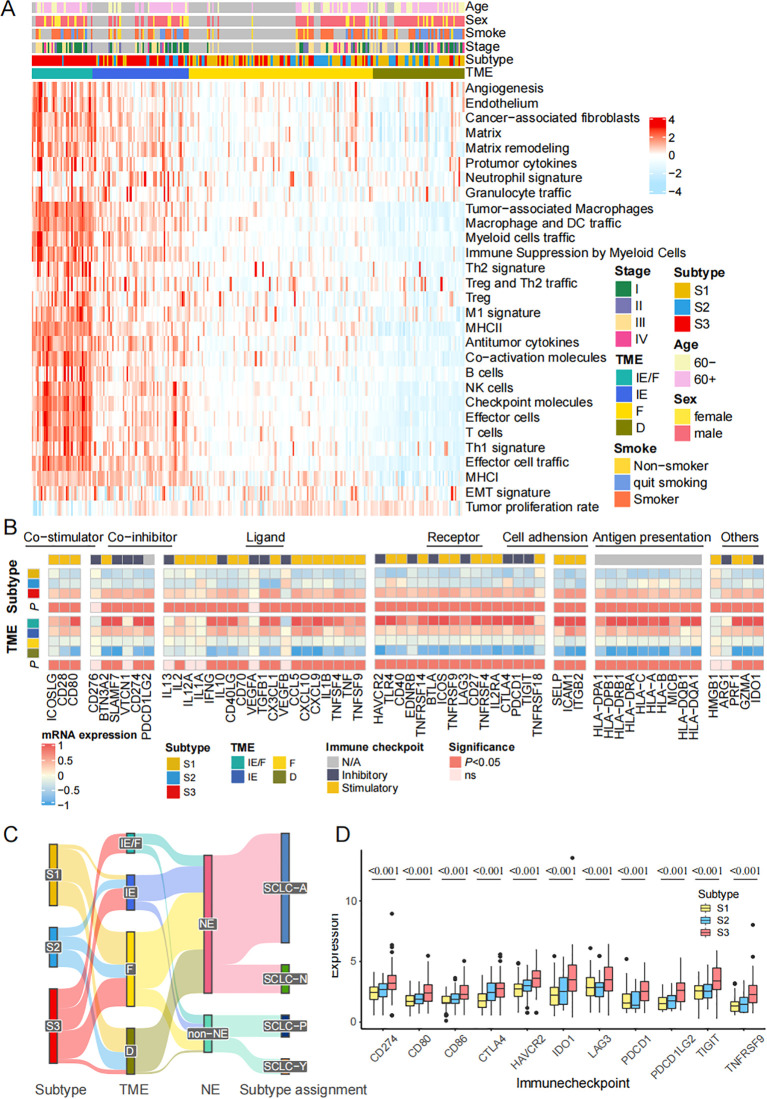
The FRGs classification is tightly correlated with TME 4 subtypes. **(A)** Heatmap shows the relative abundance of 29 functional gene expression signatures in SCLC by introducing ssGSEA algorithm. **(B)** The expression of 79 genes annotated by co-stimulator, co-inhibitor, ligand, receptor, cell adhesion, antigen presentation, others are also presented by TME subtypes. **(C)** Sankey plot shows the correlation among subtypes, TME subtypes, NE status and key transcription regulators subtypes. **(D)** Comparisons of immune checkpoint genes expression in different ferroptosis subtypes. The statistical difference of three gene clusters were compared through the Kruskal-Wallis test.

The tumor microenvironment (TME) plays a significant role in clinical outcomes and response to therapy. According to the previous study, we divided the patients into 4 TME subtypes: immune-enriched, fibrotic (IE/F), immune-enriched, non-fibrotic (IE), fibrotic (F), and immune-depleted(D) subtypes (32). We described the relative abundance of 29 functional gene expression signatures in SCLC by heatmap across these patients (**Figure 4A**). Meanwhile, the gene expression of co-stimulator, co-inhibitor, ligand, receptor, cell adhesion, antigen presentation, and others are also presented by TME subtypes (**Figure 4B**). Sankey diagram shows FRGs subtypes, TME subtypes, NE groups, and key transcription subtypes (**Figure 4C**). Comparisons of 11 immune checkpoint genes expression, including CD274, CD80, CD86, CTLA4, HAVCR2, IDO1, LAG3, PDCD1, PDCD1LG2, TIGHT, and TNFRSF9, in different ferroptosis subtypes also well distinguish the classification (**Figure 4D**). In conclusion, the FRGs classification is tightly correlated with TME 4 subtypes.

### 3.5 The FRGs classification is validated in an independent cohort

**Figure 5:**
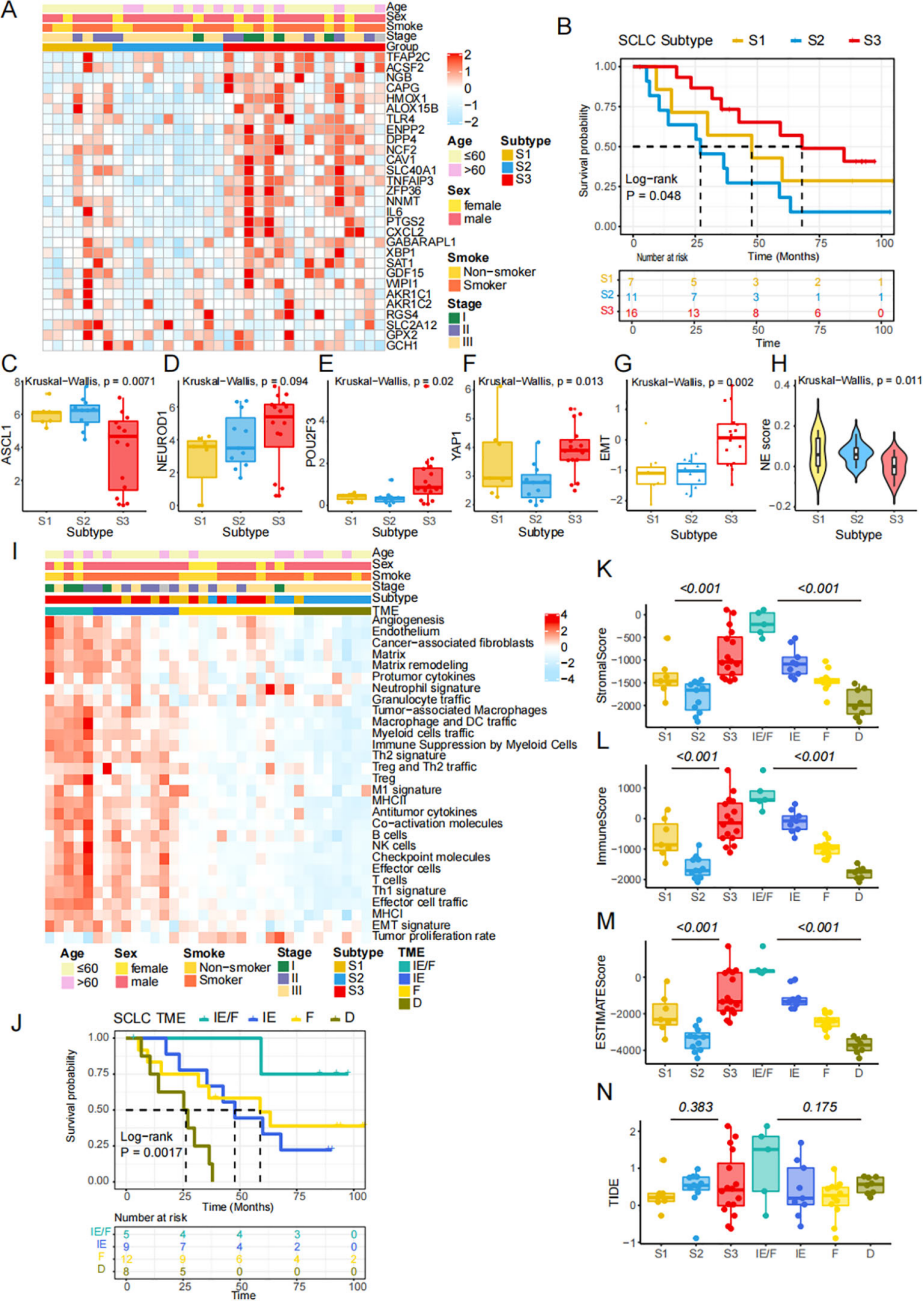
The FRGs classification is validated in an independent cohort. **(A)** Heatmap shows differential expression of NMF-selected genes. **(B)** Kaplan-Meier survival curves of SCLC patients in the three FRGs subtypes (P =0.048, Log-rank test). **(C-F)** The expression of four key transcription regulators. **(G)** The difference of EMT scores. **(H)** NE score across 3 subtypes in independent cohort. **(I)** 29 conventional pathways presenting the components in the tumor microenvironment. **(J)** Kaplan-Meier survival curves of SCLC patients in the four TME subtypes (P =0.0017, Log-rank test). **(K-M)** Stromal score, Immune score and ESTIMATE score were calculating by ESTIMATE algorithm. **(N)** TIDE score was compared across three ferroptosis subtypes and four TME subtypes.

To validate the accuracy of the classification method, we re-clustered the ferroptosis-related molecular patterns in our independent Beijing cohort of SCLC patients. We then demonstrated the correlation between the subtypes and prognosis, biological processes, and immune cell infiltration characteristics. As observed previously, the heatmap illustrates the FRGs in our data (**Figure 5A**). Kaplan-Meier survival curves of patients showed significant differences across the three subtypes (P = 0.048, Log-rank test, **Figure 5B**). Three out of four key transcription factors, including ASCL1, POU2F3, and YAP1, showed significant differences among the FRG subtypes, while NEUROD1 did not (**Figures 5C-F**). The differences in EMT score and NE score across the three subtypes remained consistent in our cohort (**Figures 5G, H**). The heatmap further demonstrates variations in the activities of pathways across the four TME subtypes (**Figure 5I**). We analyzed the immune cell infiltration of three subtypes simultaneously. The levels of macrophages M1, CD4+T and CD8+T were found to be significantly higher in S3 than in the other subtypes, which suggested that S3 had a strong immune infiltration similar to that of "hot tumors" (**Supplementary Figure S2**). This finding was consistent with **Figure 5I**. Consequently, the results indicated that S3 may benefit from immunotherapy. Survival analysis revealed differences in outcomes among the four TME groups (**Figure 5J**). We also explored the tissue content using the ESTIMATE algorithm, which showed significant differences in the Stromal score (**Figure 5K**), Immune score (**Figure 5L**), and Estimate score (**Figure 5M**). To investigate the potential for immunotherapy response, we used the TIDE method to assess the likelihood of response, as shown by the TIDE score (**Figure 5N**). In conclusion, although the limited number of our validation cohort, these results suggested the same characteristics in the public cohort and Beijing cohort based on the FRGs classification.

**Figure 6:**
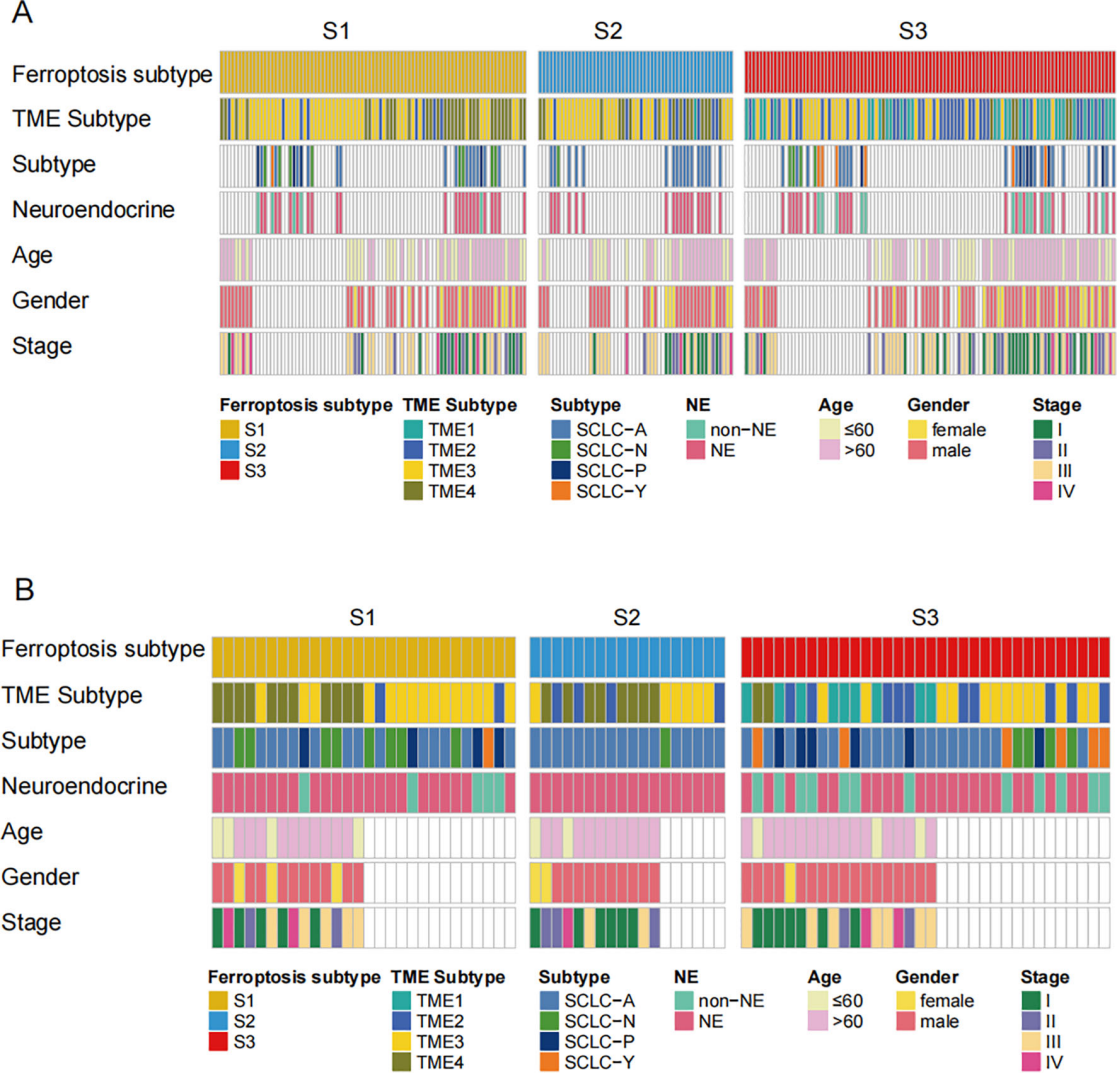
The distribution of diverse classifications in 2 cohorts. **(A, B)** Heatmap depicted the correlation between the gene subtypes and different clinicopathological features. The ferroptosis subtypes, TME subtypes, ANPY subtypes, tumor stage, Neuroendocrine state, age, gender, and age were used as patient annotations in public cohort and validation cohort.

The heatmap depicted the correlation between gene subtypes and various clinicopathological features. Ferroptosis subtypes, TME subtypes, ANPY subtypes, tumor stage, neuroendocrine state, age, and gender were used as patient annotations in the public cohort (**Figure 6A**). Similarly, the distribution of immune and molecular subtypes, along with different clinicopathological features, was analyzed in the Beijing cohort (**Figure 6B**). These heatmaps demonstrate the consistency between the public and independent cohorts, highlighting the stability and potential applicability of the FRG classification.

### 3.6 The characteristics of chemical drug prediction in FRGs subtypes

**Figure 7:**
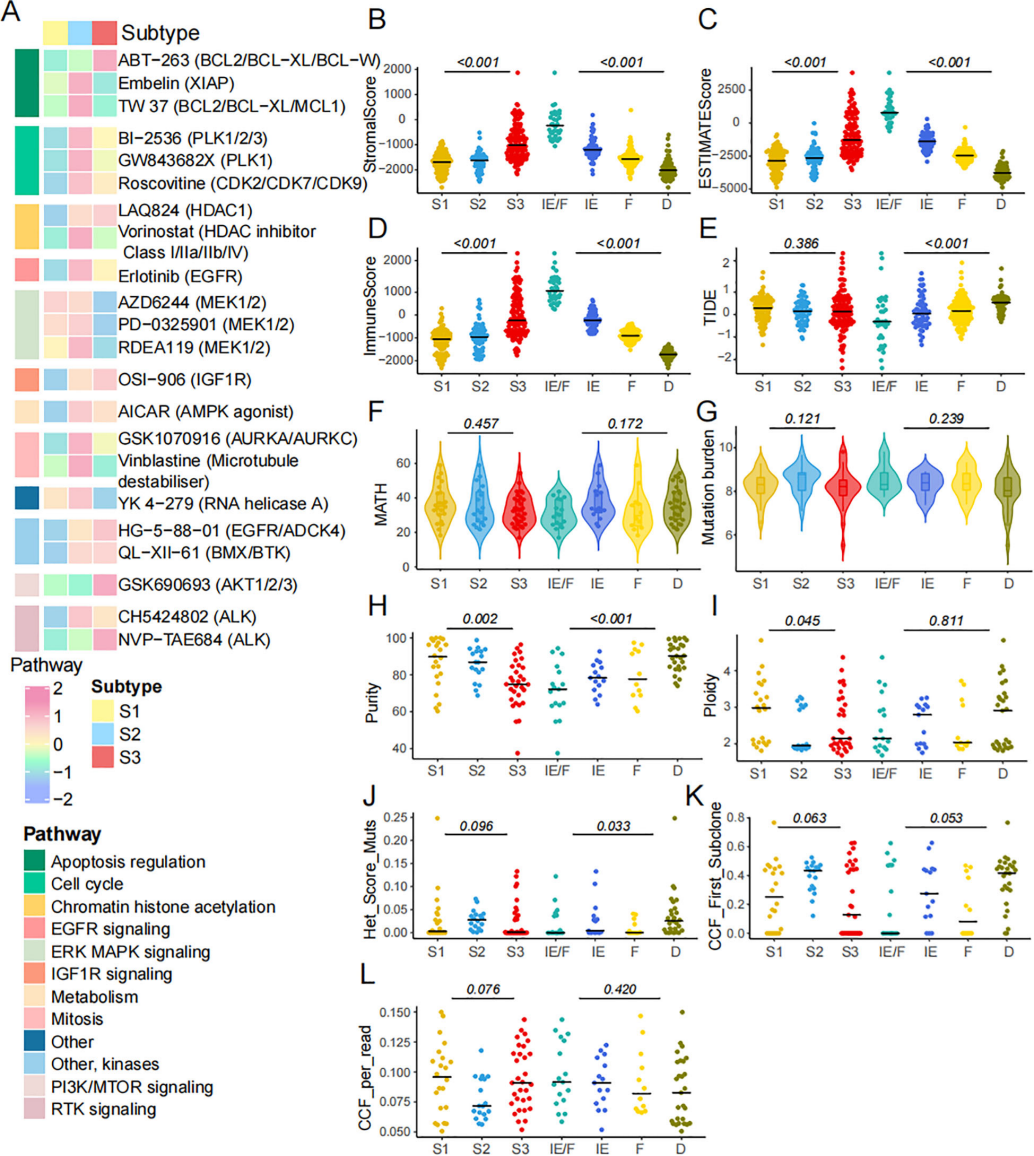
The characteristics of chemical drugs prediction in FRGs classification. **(A)** The IC50 of diverse drugs across 3 subtypes. **(B-D)** Stroma score, Immune score and ESTIMATE score across three ferroptosis subtypes and four TME subtypes. **(E)** TIDE score was compared across three ferroptosis subtypes and four TME subtypes. **(F)** The intra tumor heterogeneity is calculated by MATH. **(G-L)** Mutation burden, purity, ploidy, het mut score, CCF first score and CCF per read across 3 subtypes.

Cancer presents a range of hallmarks that offer new dimensions to describe key tumor characteristics (43). Initially, we acquired data from the GDSC database to explore potential chemical drugs. These drugs primarily target multiple pathways, including apoptosis regulation (**Figure 7A**). Previous studies have demonstrated that immune components, tumor heterogeneity, mutation burden, tumor purity, and subclonal architectures may predict therapeutic responses. To investigate potential immunotherapy responses in SCLC subtypes, we performed SubMap analysis by comparative gene expression profiling between a publicly available cohort of 47 melanoma patients treated with anti-PD-1 and anti-CTLA-4 and the SCLC ferroptosis subtype. The S3 subgroup exhibited significant similarity to melanoma responders, suggesting a promising likelihood of sensitivity to anti-PD-1 therapy (Bonferroni-corrected p = 0.048, **Supplementary Figure S3** A; nominal p = 0.004, **Supplementary Figure S3B**). These findings suggested a potential mechanistic role for ferroptosis-related mRNAs in modulating immunotherapy efficacy. To assess these factors, we compared the ESTIMATE score, immune score, and stromal score across the three FRG subtypes and four TME subtypes, revealing significant differences (**Figures 7B-D**). However, no differences were observed in the TIDE score across the three FRG subtypes, suggesting that the FRGs do not correlate with immunotherapy response (**Figure 7E**). Next, we explored intra-tumor heterogeneity in the cohort using MATH (**Figure 7F**) and also examined mutation burden (**Figure 7G**). Neither heterogeneity nor mutation burden showed a correlation with FRG classification. In terms of tumor purity, we found differences across the FRG and TME subgroups (**Figure 7H**). Furthermore, we investigated various subclonal architectures of SCLC, such as ploidy and cancer cell fraction (CCF). We observed that ploidy was higher in S1 (**Figure 7I**). However, there were no significant differences in the association between FRG subtypes and the heterogeneity mutation score (Het Mut Score), CCF first score, or CCF per read (**Figures 7J-L**). In conclusion, the FRG subtypes hold significant potential for guiding drug selection in SCLC therapy.

## 4 Discussion

SCLC accounts for approximately 15% of all lung cancers. It is characterized by an exceptionally high proliferative rate, a strong predisposition for early metastasis, and poor prognosis, highlighting the urgent need for more effective therapies. Rudin et al. constructs a novel model of SCLC subtypes defined by differential expression of four key transcription regulators: ASCL1, NeuroD1, YAP1, and POU2F3 according to SCLC primary human tumors, patient-derived xenografts, cancer cell lines, and genetically engineered mouse models (2). Meanwhile, Bagaev et al. identified four TME subtypes that were conserved across various cancers and correlated with immunotherapy responses, including in SCLC (32). However, there are still some limitations that both studies in this issue found some inconsistencies in the findings of the proposed molecular subtypes. That shows one essential need for novel classification.

In the past decade, ferroptosis, an iron-dependent form of regulated cell death driven by excessive lipid peroxidation, has been implicated in the development and therapeutic responses of various types of tumors. However, as an important biological behavior in cell death, ferroptosis has never been linked to SCLC progression. In this study, we develop a model of distinct subtypes defined by the relative expression of FRGs. The landscape of genetic variation presented the high heterogeneity of genetic and expressional alteration landscape in ferroptosis-related genes between normal and SCLC samples, indicating that the expression imbalance of ferroptosis played a crucial role in the SCLC occurrence and progression. We determined three distinct FRG subtypes in SCLC according to the expression of FRGs. The three FRG subtypes presented significant survival differences.

Previous studies reveal that the activating Notch signaling tumor cells are slowly growing and often chemo-resistant. Accordingly, the Notch is considered a tumor suppressor in SCLC (44). Based on the inhibitory activity of DLL3 on the Notch pathway or the inactivating mutations in Notch pathway genes, Notch is frequently inactivated in SCLCs with a high NE expression profile. Meanwhile, the activation of Notch signaling in a preclinical SCLC mouse model strikingly reduced the number of tumors and extended the survival of the mutant mice (18). In neuroendocrine cells, MYC activates Notch to dedifferentiate tumor cells, promoting a temporal shift in SCLC from ASCL1+ to NEUROD1+ to YAP1+ states (45). Interestingly, we found that the activity of the notch pathway, notch ligand pathway, and MYC pathway were different in diverse subtypes. Given that the majority of tumors express a neuroendocrine program that integrates neural and endocrine properties, we tried to find the connection between our classification and the characteristics of neuroendocrine. The activity of NE was also well distinguished in the different clusters. Furthermore, the EMT score also showed a specific level among these subtypes. The above findings indicated a pronounced divergence in the subtypes based on the expression of FRGs.

The TME subtypes correlate with patient response to immunotherapy in multiple cancers, with patients possessing immune-favorable TME subtypes benefiting the most from immunotherapy (9, 46).In particular, cell-to-cell communication in the TME has a major impact on the efficacy of immunotherapy and prognosis (47). Regarding the inclusion of malignant and microenvironment components, TME subtypes act as a generalized immunotherapy biomarker across many cancer types, including SCLC. Therefore, we also tried to describe the distribution of TME subtypes in our cohort. Then, the expression of Immune checkpoint genes is explored in the TME subtypes and key transcription subtypes. In summary, we found that the FRGs-based classification is tightly correlated with TME 4 clusters, indicating the FRGs classification might be indicating the important role of tumor microenvironment. To validate the findings, we adopted one new independent cohort in Beijing including 34 SCLC patients. Most of the FRGs were predominantly expressed in the S3 subtype which had the best prognosis. The expression of four key transcription regulators was well distinguished in the 3 subtypes. Meanwhile, the EMT score and NE score are different. Interestingly, the proposition of the TME component in the Beijing cohort is similar to the public cohort. Survival analysis in the TME classification is significant. At the same time, stromal score, immune score, and ESTIMATE score are different in FRGs subtypes. In summary, we find the characteristics in the validation cohort are similar in the training set.

Next, we explored the relationship between the IC50 values of various drugs and the classification using the GDSC database. Interestingly, the IC50 values were higher in the S2 subtype, suggesting lower sensitivity to chemical therapies and indicating worse outcomes in this group. We also evaluated the immune score, stromal score, and ESTIMATE score in the training set, finding significant differences across subtypes. However, no differences were observed in the TIDE score, which is typically used to evaluate immunotherapy response based on RNA sequencing data. Previous studies have shown that tumor heterogeneity serves as a biomarker for therapy efficiency, as it fuels resistance. Therefore, an accurate assessment of tumor heterogeneity is essential for developing effective therapies (48). To explore this, we assessed intra-tumor heterogeneity using the MATH score (37). SCLCs are prone to acquiring diverse genetic alterations as they undergo evolution. Genomic profiling of SCLC reveals extensive chromosomal rearrangements and a substantial mutation burden (49). Our findings indicated no disparities in heterogeneity and mutation burden. Dvir Aran et al. demonstrate that lower-purity samples can hinder the efficacy of precision medicine by influencing genomic data (50). Additionally, we calculated the purity across the samples and found significant differences between groups. Aneuploidy is a ubiquitous feature of human tumors and may function as an under-explored cause of therapy failure (51, 52). While, the characteristic and subclonal architecture of cancer, including het mut score, CCF first score, and CCF per read are not significant. At present, extensive-stage SCLC is treated with immunotherapy combination in the first-line treatment, but the proportion of effective long-term survival is low. FRG subtype may be effective in aiding the identification of patients for long-term benefit. In addition, a heterogeneous strategy to improve survival may be available for those S2 patients with poor prognosis.

Our study has some limitations. The results were validated in the independent Beijing cohort, but the sample size was limited, and a larger cohort is needed in the future. The treatment information of patients in both the public and Beijing cohorts was unknown, which may have impacted the accuracy of the subtypes and prognosis prediction. It is imperative to restrict enrollment in an independent cohort, particularly to predict the benefit of immunotherapy using FRGs-based subtypes. Furthermore, we perform a drug sensitivity analysis, but more in-depth ex vivo and *in vivo* research is necessary.

## 5 Conclusion

This was the first characterization of the landscape of ferroptosis regulators—including their molecular characteristics, immuno-oncology features, and clinical relevance. Our data highlight the importance of FRGs on cancer pathogenesis and shaping of TME and lay a rational foundation and buttress for developing therapeutic strategies for targeting ferroptosis in patients with SCLC.

## Data Availability

The datasets presented in this study can be found in online repositories. The names of the repository/repositories and accession number(s) can be found in the article/**Supplementary Material**.

## Supplementary material

Supplementary Figure 1 The study of workflow.

Supplementary Figure 2 Three subtypes of TME cell composition.

Supplementary Figure 3 SubMap analysis showed the similarity of gene expression profiles between ferroptosis-associated subtypes and melanoma patients with anti-PD-1 and anti-CTLA4 treatment; **(A)** Bonferroni-corrected p< 0.05; **(B)** nominal p<0.05.
