# Supplementary figures and images for: Molecular subtypes based on ferroptosis-related genes and tumor microenvironment infiltration characterization in small cell lung cancer
Source: Front Immunol. 2025 May 13;16:1574434. doi: 10.3389/fimmu.2025.1574434 (PMC12106331; doi:10.3389/fimmu.2025.1574434)

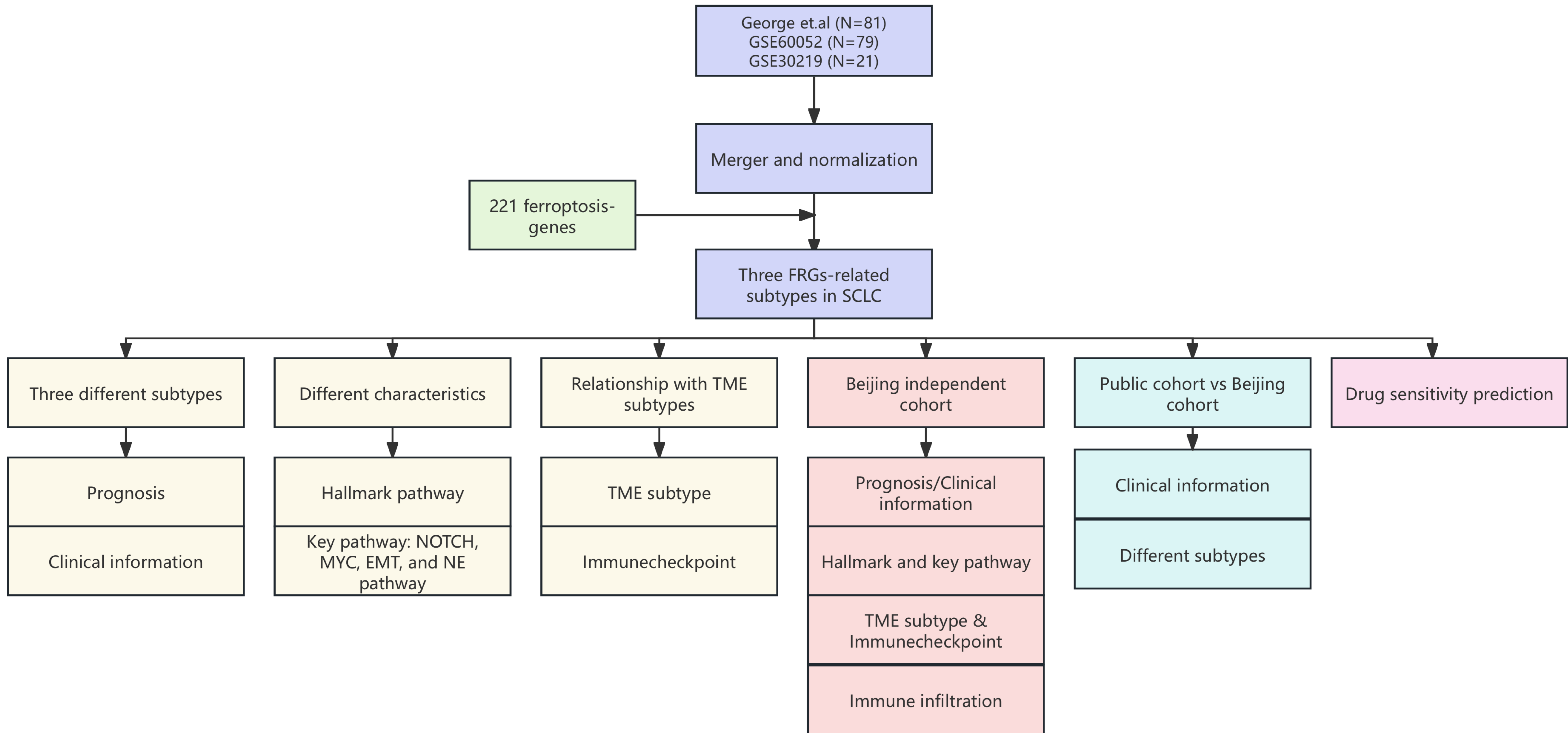

Supplement: Supplementary Figure 1 — The study of workflow. [file Presentation1.pdf]

# TME Cell composition

Group C1 C2 C3

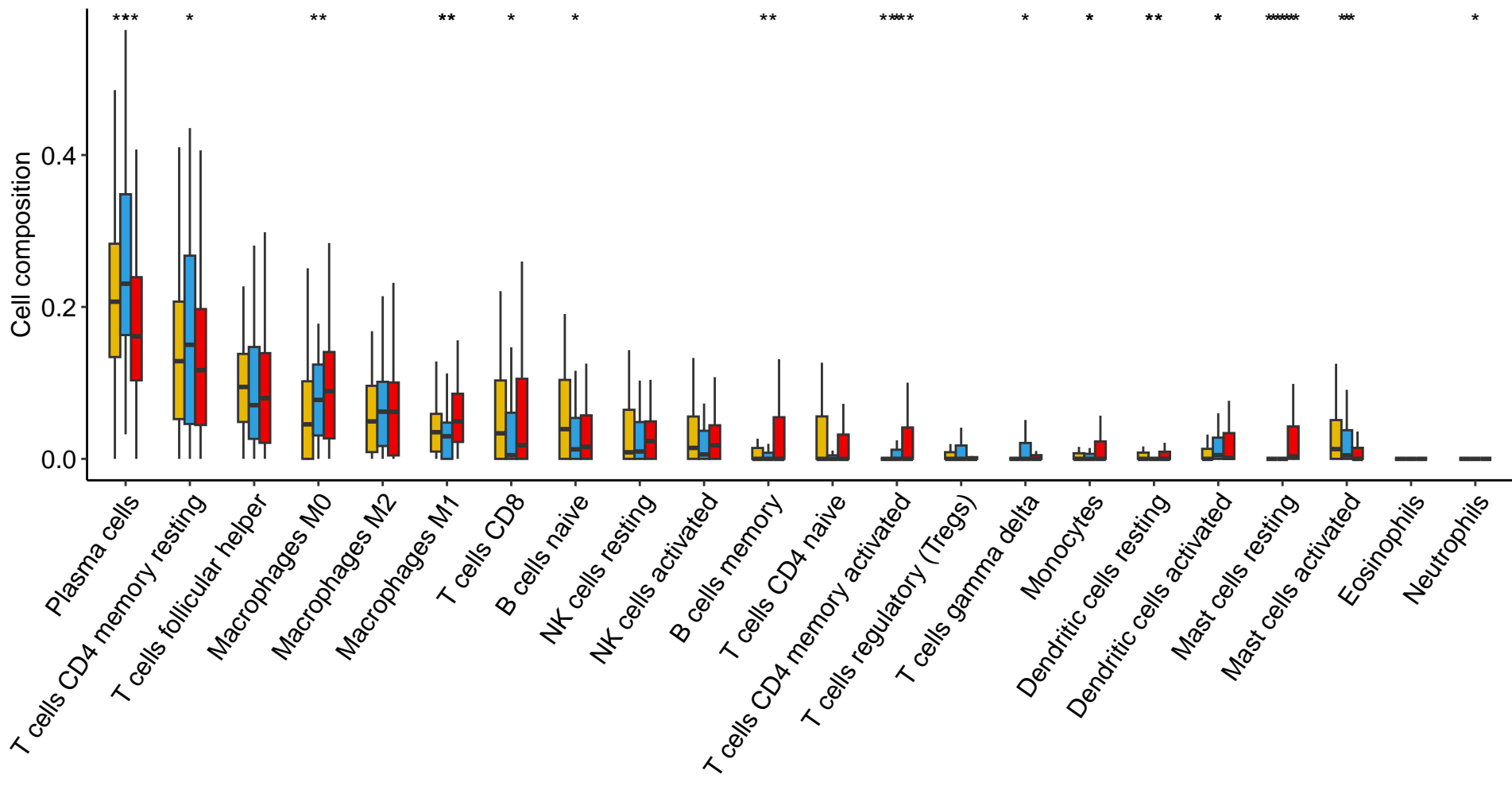

Supplement: Supplementary Figure 2 — Three subtypes of TME cell composition. [file Presentation2.pdf]

A

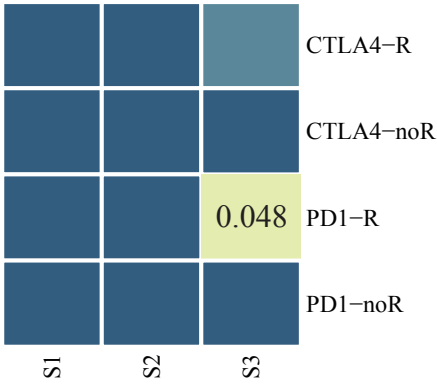

B

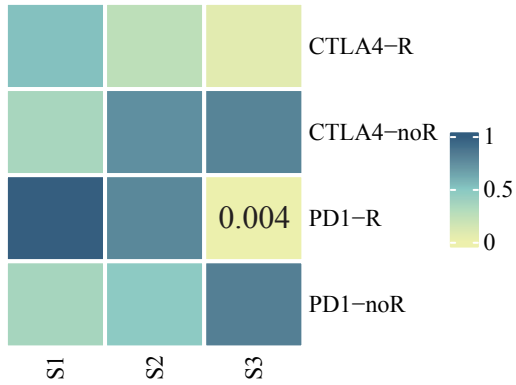

Supplement: Supplementary Figure 3 — SubMap analysis showed the similarity of gene expression profiles between ferroptosis-associated subtypes and melanoma patients with anti-PD-1 and anti-CTLA4 treatment; (A) Bonferroni-corrected p< 0.05; (B) nominal p<0.05. [file Presentation3.pdf]
